# Demystifying Online instruction in Libraries: People, Processes and Tools

**DOI:** 10.29173/jchla29532

**Published:** 2021-04-02

**Authors:** Jack Young

**Affiliations:** Health Sciences Librarian, McMaster University, Hamilton, ON, Canada

Turnbow B, & Roth A. **Demystifying Online instruction in Libraries: People, Processes and Tools**. Chicago: ALA Editions; 2020. Softcover: 104 p. ISBN: 978-0-8389-1937-8. Price: USD $59.99. Available from: https://www.alastore.ala.org/content/demystifying-online-instruction-libraries-people-process-and-tools

Almost all libraries engage in some form of online instruction. As library service models have shifted in response to the COVID-19 pandemic, this type of instruction has become even more prevalent. Despite the widespread adoption of online instruction, few libraries have invested the time and resources necessary for ongoing success in this field. A haphazard approach to online learning leads to inefficient workflows and learning objects that are poorly suited to our users’ needs.

Written by Dominque Turnbow and Amanda Roth, *Demystifying Online Instruction in Libraries: People, Process, and Tools*, explores the specialized processes and expertise necessary for sustainable online instruction in libraries. By advocating for the distinct roles of Instructional Designers and Instructional Technologists within the library, the authors make a strong argument for increased specialization in the practice of online learning. In a simple and concise style, the book breaks down the key processes that go into developing successful online materials and serves as an excellent primer for instruction librarians and library managers alike.

Not intended to be a deep dive into any one aspect of the online instruction process, the book succinctly highlights a wide range of core concepts, theories, and practices across its eight chapters. The opening chapter clearly defines the roles of Instructional Designer and Instructional Technologist and explores strategies for advocating for such skill sets within your library. While the authors suggest that it would be ideal to create two distinct positions for these roles (as is the case at their home institution of UC San Diego,) they acknowledge that this is not realistic for every library and provide useful tips for building up these skills in existing library staff. Across the remaining seven chapters, readers are offered an effective introduction to topics ranging from design theory, to project management, to specific instructional technologies.

It would be easy to get bogged down in the details with so much content to cover, but the authors strike a good balance between theory and practice. Evenly dispersed sections called “ID in Action” allow the authors to share their own experiences with the topic in question through real-world projects they have worked on. In addition, a substantial appendices section provides a valuable trove of document templates that could be adapted to your own library’s needs.

As a librarian who juggles multiple responsibilities in addition to online instruction, I occasionally felt a bit overwhelmed by the book’s recommended workflow. I cannot imagine engaging in the all the planning, consultation, assessment, and documentation required for each project while still having time for my other job responsibilities. Ultimately, though, the fact that I felt overwhelmed serves to underline the authors’ primary thesis: that proper online instruction cannot just be something tacked onto an existing librarian’s portfolio; that to engage in meaningful education online, libraries must invest the necessary resources (human, temporal, and financial). Although the book does not touch on the COVID-19 pandemic explicitly, this message seems even more vital as libraries continue to serve a growing population of remote learners.

I would recommend *Demystifying online instruction in libraries* to a wide range of library workers. Library school students and early career librarians will benefit from its straightforward introduction to instructional design principles, while more experienced instruction librarians will pick out useful tips and tricks from its multiple real-world examples. Finally, library managers looking to increase the impact of their library’s online instruction efforts will come away with a clear image of a well-organized instruction department. At just over 100 pages, the reader can receive a fairly thorough introduction to online instruction over the course of an afternoon. While the $60USD price tag may seem a bit steep for such a slim volume, this is the kind of book that you will find yourself returning to again and again, as evidenced by the heavily dog-eared copy on my own shelf.

